# Prognostic Markers in Hospitalized COVID-19 Patients: The Role of IP-10 and C-Reactive Protein

**DOI:** 10.1155/2022/3528312

**Published:** 2022-02-28

**Authors:** Manuela Rizzi, Martina Costanzo, Stelvio Tonello, Erica Matino, Francesco Giuseppe Casciaro, Alessandro Croce, Eleonora Rizzi, Erika Zecca, Anita Pedrinelli, Veronica Vassia, Raffaella Landi, Mattia Bellan, Luigi Mario Castello, Rosalba Minisini, Venkata Ramana Mallela, Gian Carlo Avanzi, Mario Pirisi, Daniele Lilleri, Pier Paolo Sainaghi

**Affiliations:** ^1^Rheumatology Unit, AOU “Maggiore della Carità”, Novara 28100, Italy; ^2^Department of Internal Medicine and COVID-19 Unit, AOU “Maggiore della Carità”, Novara 28100, Italy; ^3^Department of Translational Medicine, Università del Piemonte Orientale (UPO), Novara 28100, Italy; ^4^Division of Emergency Medicine and COVID-19 Sub-Intensive Unit, AOU “Maggiore della Carità”, Novara 28100, Italy; ^5^CAAD, Center for Autoimmune and Allergic Diseases, Università del Piemonte Oreintale (UPO), Novara, Italy; ^6^Unit of Microbiology and Virology, IRCCS Policlinico San Matteo Foundation, Pavia 27100, Italy

## Abstract

**Background:**

SARS-CoV-2 is responsible for COVID-19, a clinically heterogeneous disease, ranging from being completely asymptomatic to life-threating manifestations. An unmet clinical need is the identification at disease onset or during its course of reliable biomarkers allowing patients' stratification according to disease severity. In this observational prospective cohort study, patients' immunologic and laboratory signatures were analyzed to identify independent predictors of unfavorable (either death or intensive care unit admission need) or favorable (discharge and/or clinical resolution within the first 14 days of hospitalization) outcome.

**Methods:**

Between January and May 2021 (third wave of the pandemic), we enrolled 139 consecutive SARS-CoV-2 positive patients hospitalized in Northern Italy to study their immunological and laboratory signatures. Multiplex cytokine, chemokine, and growth factor analysis, along with routine laboratory tests, were performed at baseline and after 7 days of hospital stay.

**Results:**

According to their baseline characteristics, the majority of our patients experienced a moderate to severe illness. At multivariate analysis, the only independent predictors of disease evolution were the serum concentrations of IP-10 (at baseline) and of C-reactive protein (CRP) after 7 days of hospitalization. Receiver-operating characteristic (ROC) curve analysis confirmed that baseline IP − 10 > 4271 pg/mL and CRP > 2.3 mg/dL at 7 days predict a worsening in clinical conditions (87% sensitivity, 66% specificity, area under the curve (AUC) 0.772, *p* < 0.001 and 83% sensitivity, 73% specificity, AUC 0.826, *p* < 0.001, respectively).

**Conclusions:**

According to our results, baseline IP-10 and CRP after 7 days of hospitalization could be useful in driving clinical decisions tailored to the expected disease trajectory in hospitalized COVID-19 patients.

## 1. Introduction

In December 2019, many cases of pneumonia of unknown origin were reported in Wuhan (Hubei province, China). Since then, this illness rapidly spread across China and all over the world. The causative agent of this new disease has been identified in SARS-CoV-2, a positive, single-stranded RNA virus with a genome length of less than 30 kb, belonging to the same *β*-coronavirus genus of SARS-CoV and MERS-CoV [1–4]. SARS-CoV-2 virus enters host's cells by binding the angiotensin-converting enzyme 2 (ACE2) expressed in nasal epithelium, low airways, and lungs [4–7]. This new viral agent is characterized by a high interhuman transmission rate: according to the current evidence, World Health Organization (WHO) reports that respiratory droplets and direct contact represent the most common routes of infection [4–6, 8–10].

Patients affected by COVID-19 show a wide range of clinical manifestations, ranging from a nearly asymptomatic or mild flu-like condition to severe interstitial pneumonia and acute respiratory distress syndrome. It is well-recognized that severe clinical manifestations depend not only on viral infection but also on a heavy inflammatory response [1–4, 11, 12]. Pathogen recognition by antigen presenting cells activates both innate and adaptive immune cells to produce large amounts of proinflammatory mediators, in some cases leading to systemic spread of the aberrant immune response leading—in the most severe cases—to multiple organ failure and death [2, 13–15].

Reliable biomarkers allowing clinicians to identify, at an early stage, those patients whose condition will deteriorate would facilitate patients' stratification into risk groups for optimal resources allocation. To meet this need, many clinical research groups, including ours, analyzed their case series, identifying several demographic (i.e., age, gender, and comorbidities) and laboratory (i.e., CRP (C-reactive protein), IL-6, D-dimer, and creatinine) parameters that could be used as prognostic biomarkers [14, 16–21]. Unfortunately, the retrospective design of these studies has many potential biases.

The present prospective, observational monocentric study is aimed at investigating cytokine, chemokine, and growth factor expression in an Italian cohort of SARS-CoV-2 positive patients, by using a large and unbiased multiplex quantification approach. To this purpose, patients were studied at the time of hospital admission and along their hospital stay, during which they received per protocol corticosteroids and heparin, to identify possible biomarkers able to predict disease severity and evolution.

## 2. Methods

### 2.1. Patients

We performed a prospective, observational cohort study during the third wave of COVID-19 epidemic in Italy: 139 consecutive patients were enrolled in non-ICU (intensive care unit) wards (including high-dependency/subintensive units) of “Maggiore della Carità” University Hospital in Novara, Italy, between January and May 2021. This study is part of the large multicenter observational study “BIAS” (Baseline Immunity status effect on sArs-cov2 presentation and evolution: comparison between immunocompetent and immunocompromised patients). The study protocol was approved by the local ethical committee (CE 7/21) and was conducted in strict accordance with the Declaration of Helsinki. Patients were selected according to precise inclusion and exclusion criteria and were asked to give a written consent. To be eligible for the study, patients should be adults (>18 years), hospitalized for confirmed SARS-CoV-2 infection (molecular RT-PCR or quick test positivity), with clinical symptoms not exceeding 12 days. Patients needing an immediate ICU admission or with a very severe clinical presentation (stage V renal failure, severe oncological condition) were excluded.

All patients meeting inclusion criteria for the study received treatment according to the “Maggiore della Carità” Hospital internal protocol. Specifically, all patients received oxygen supplementation, corticosteroids, and low molecular weight heparin (LMWH) unless contraindicated.

### 2.2. Endpoints Definition

The expected endpoints were the following: (1) identification of biomarkers predicting at baseline and after 7 days of hospitalization an adverse disease evolution (death or ICU admission); (2) identification of clinical or immunological biomarkers predicting at baseline and after 7 days of hospitalization a rapid clinical recovery (discharge from hospital and/or National Early Warning Score 2 (NEWS2) ≤ 2 for at least 24 hours within the first 14 days of hospitalization).

### 2.3. Blood Sample Collection

Blood samples for routine analysis and for multiplex quantifications were collected by venous puncture using EDTA as anticoagulant at baseline (*t*0) and after 7 days of hospitalization (*t*7). Blood fractions were immediately separated by centrifugation and stored at -80°C until the time of analysis.

### 2.4. Routine Laboratory Evaluation

For each patient, routine laboratory studies included a complete blood cell count, a common biochemistry panel (i.e., creatinine, alanine aminotransferase (ALT), and aspartate aminotransferase (AST)), as well as inflammatory markers (i.e., CRP and ferritin) and markers of coagulation and fibrinolysis (including D-dimer).

### 2.5. Multiplex Analysis

Twenty-seven plasma cytokines, chemokines, and growth factors were analyzed using the Bio-Plex Pro Human Cytokine 27-plex panel (Bio-Rad Laboratories Inc, Hercules, CA, USA) following the manufacturer instructions. Prior to quantification, plasma samples were diluted 1 : 4 in standard diluent (provided by the manufacturer). By using such panel, serum samples were screened for interleukin (IL)-1*β*, IL-1RA, IL-2, IL-4, IL-5, IL-6, IL-7, IL-8, IL-9, IL-10, IL-12, IL-13, IL-15, IL-17, eotaxin, basic fibroblast growth factor (FGF), granulocyte-colony stimulating factor (G-CSF), granulocyte-macrophage colony stimulating factor (GM-CSF), interferon (IFN)-*γ*, IFN-*γ* induced protein-10 (IP-10), monocyte chemoattractant protein-1 (MCP-1), macrophage inflammatory protein (MIP)-1 *α*/*β* (MIP-1*α*, MIP-1*β*), platelet-derived growth factor (PDGF), RANTES (regulated on activation normal T-cell expressed and secreted), tumor necrosis factor *α* (TNF-*α*), and vascular endothelial growth factor (VEGF) concentrations. Fluorescent signals were recorded using a Bio-Plex 200 System instrument and analyzed using the Bio-Plex Manager Software (Bio-Rad Laboratories Inc, Hercules, CA, USA). The software fitted samples' median fluorescence intensity (MFI) values versus standards' MFI and converted it to concentration (pg/mL) by applying a five-parameter logistic regression (as suggested by the manufacturer).

### 2.6. Data Collection and Statistical Analysis

A web-based database (RedCap platform) was used to store and manage clinical and laboratory data of each patient (demographics, clinical parameters, therapeutic schedule, and laboratory parameters). Clinical and routine laboratory data were collected by carefully reviewing medical records of each patient, starting from the time of admission (baseline, *t*0), until discharge (or for a maximum of 28 days) or study exit (death or ICU admission). Data extracted from the RedCap database and multiplex quantifications underwent univariate and multivariate statistical analysis to evaluate their significance toward the expected endpoints. For continuous variables, the measures of central tendency and dispersion chosen were medians and interquartile range (IQR). Categorical variables were presented as frequencies (percentage). Variables were compared with the Mann-Whitney *U* test (continuous variables) or Pearson *χ*^2^ test (categorical variables). Data obtained from univariate analysis were used to build multiple regression models. Receiver-operating characteristic (ROC) curves were drawn to identify the prognostic cut-off for clinical parameters according to the corresponding AUC (area under the curve) score. The threshold chosen to indicate statistical significance was 0.05 (two-tailed). Statistical tests were performed either with the software package Statistica for Windows, release 12 (TIBCO Software Inc., Palo Alto, CA, USA) or the MedCalc® Statistical Software, version 20.014 (MedCalc Software Ltd., Ostend, Belgium).

## 3. Results

From January until May 2021, 139 SARS-CoV-2-positive patients admitted to non-ICU wards of “Maggiore della Carità” Hospital in Novara meeting the study inclusion criteria were enrolled to the present study: 86 were males (61.9%), and 53 (38.1%) were females.

The most common symptoms at hospital admission (*t*0) were dyspnea (62.6%) and dry cough (38.9%). Moreover, among the 139 patients, 83 started a COVID-19-related treatment before hospital admission (corticosteroids (52.5%), azithromycin (35.2%), and heparin (30.9%)). The majority of the enrolled patients at the time of hospital admission showed a moderate (74.1%) or severe (6.5%) respiratory failure (we defined the respiratory failure as moderate when 100 ≤ PiO_2_/FiO_2_ < 200 and severe when PiO_2_/FiO_2_ < 100). Consistently median NEWS2 score recorded at the time of admission in our cohort was 5 (IQR: 4-6), a value indicating a potentially serious clinical deterioration in patient's conditions, requiring a close clinical monitoring [22]. The detailed demographical and baseline (*t*0) clinical description of the selected population is shown in Table 1.

Among the 139 patients initially enrolled to the study, 29 died during hospital stay or were transferred to ICU, while of the remaining 110 patients, 91 were discharged or reached a NEWS2 ≤ 2 for at least 24 hours within the first 14 days of hospitalization.

### 3.1. Outcomes

1. We compared data obtained at baseline and at 7 days from the 29 patients who died or were transferred to ICU during hospital stay to those obtained from all the other patients

Univariate statistical analysis is shown in Tables 2 and 3. At baseline, patients evolving toward a more severe form of the disease showed lower platelet and lymphocyte counts and glomerular filtration rates, in addition to higher RDW-CV (red cell distribution width–coefficient of variation), creatinine, LDH (lactic dehydrogenase), ferritin, D-dimer, IL-6, IL-8, IP-10, and MCP-1 values (Table 2). After 7 days of hospital stay, patients with a worse disease evolution showed a statistically significant lower level in lymphocyte and platelet counts as well as in TNF-*α* levels and an increment in neutrophil to lymphocyte ratio, CRP, LDH, erythrocyte sedimentation rate, troponin I, ferritin, D-dimer, G-CSF, IFN-*γ*, IL-6, IL-8, IP-10, MCP-1, and MIP-1*α* values (Table 3).

Parameters being significant (*p* < 0.05) at univariate analysis were used to build multivariate analysis models to identify independent predictors of outcome (Tables 4 and 5). Higher RDW-CV, IP-10, and D-dimer values and lower platelet count at baseline predicted a worsening in clinical conditions (i.e., death or need to ICU admission) (Table 4) also after correction for demographic and COVID-19 severity variables (Table 5), while after 7 days of hospitalization, the only biomarker with prognostic significance was the increase in CRP levels (Table 6) again confirmed after correction for demographic and COVID-19 severity variables (multivariate analysis: CRP *β* 0.3372, *p* = 0.0049; sex *β* -0.1960, *p* = 0.0779; PiO_2_/FiO_2_*β* -0.1776, *p* = 0.1664; NEWS2 *β* 0.1302, *p* = 0.2975; age *β* 0.0216, *p* = 0.8701).

(2) We evaluated which biomarkers were useful to early identify patients who had a faster clinical recovery (hospital discharge or NEWS2 ≤ 2 for at least 24 hours within 14 days of hospitalization) with respect to all the other patients

Univariate statistical analysis (Tables 7 and 8) highlighted a significant alteration in some parameters between the two considered populations. In particular, it has been observed that, at baseline, patients with a faster clinical resolution showed a reduction in RDW-CV, creatinine, CRP, neutrophil to lymphocyte ratio, troponin I, D-dimer, IL-4, IL-6, IL-8, IL-10, IP-10, MCP-1, and MIP-1*α* in addition to an increase in lymphocytes and platelets count and glomerular filtration rate (Table 7). At *t*7, indeed, it has been observed that patients with a more favorable prognosis showed an increase in platelets and lymphocytes counts, glomerular filtration rate, ALT, and albumin, in addition to a decrease in neutrophil to lymphocyte ratio, RDW-CV, neutrophil count, CRP, LDH, erythrocyte sedimentation rate, troponin I, D-dimer, G-CSF, IFN-*γ*, IL-6, IL-8, IP-10, MCP-1, and MIP-1*α* (Table 8).

Again, multivariate models were built to identify the laboratory findings better identifying patients with a faster recovery (hospital discharge or NEWS ≤ 2 within 14 days of hospitalization) (Tables 9 and 10). Such analysis highlighted that patients with a more favorable prognosis showed lower IP-10 and neutrophil to lymphocyte ratio values and higher glomerular filtration rate at the time of hospital admission (*t*0) (Table 9). Interestingly, after correction for age, gender, and severity of clinical presentation (NEWS2 score and PiO_2_/FiO_2_ at baseline), only low IP-10 values appeared to be related to a favorable prognosis (Table 10). After 7 days of hospitalization, indeed, the only laboratory finding with prognostic significance for a more positive outcome was a low CRP value (Table 11) even after correction for age, gender, and severity of the disease (NEWS2 and PiO_2_/FiO_2_ at 7 days) (multivariate analysis: CRP *β* 0.4786, *p* = 0.0001; NEWS2 *β* -0.2143, *p* = 0.0602; PiO_2_/FiO_2_*β* 0.2175, *p* = 0.0622; age *β* -0.1319, *p* = 0.2723; sex *β* -0.0430, *p* = 0.6647).

**Figure 1:**
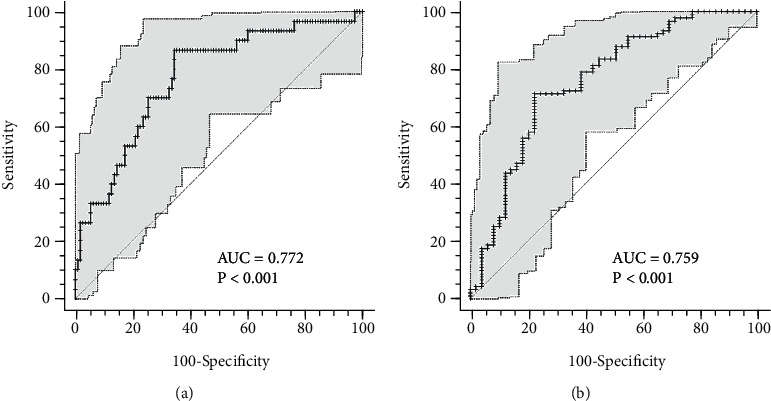
ROC curves for IP-10 at the time of hospital admission. (a) ROC curve predicting severe disease evolution; (b) ROC curve predicting shorter hospital stay. AUC : area under the curve.

**Figure 2:**
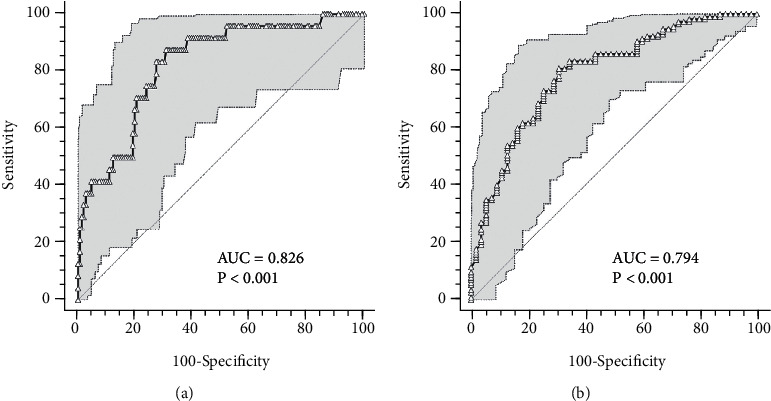
ROC curves for CRP after 7 days hospitalization. (a) ROC curve predicting severe disease evolution; (b) ROC curve predicting shorter hospital stay. AUC: area under the curve.

**Figure 3:**
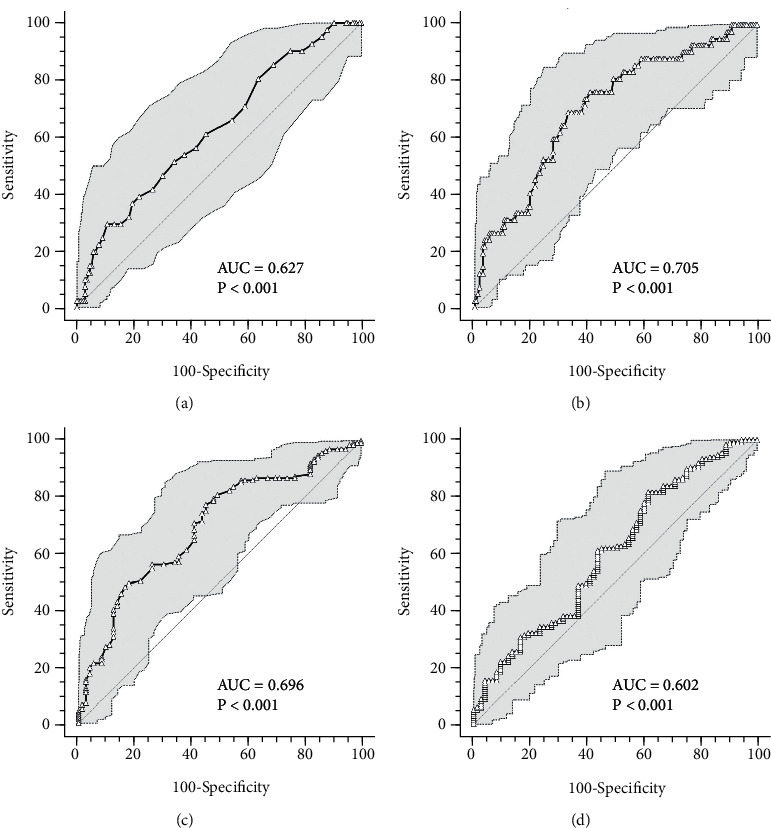
ROC curves for RDW-CV, platelet count, glomerular filtration rate, and neutrophil to lymphocyte ratio at the time of hospital admission. (a) RDW-CV ROC curve predicting severe disease evolution; (b) platelet count ROC curve predicting severe disease evolution; (c) glomerular filtration rate ROC curve predicting a shorter hospital stay; (d) neutrophil to lymphocyte ratio ROC curve predicting a shorter hospital stay. AUC: area under the curve.

Finally, to calculate the accuracy of the identified prognostic biomarkers, ROC curves were drawn, and cut-off values were identified according to the corresponding AUC (Figures 1–3). For IP-10 measured at baseline, the best cut-off value (0.7 < AUC ≤ 0.9) was 4271 pg/mL: IP-10 higher than 4271 pg/mL predicted a worsening in clinical conditions (87% sensitivity, 66% specificity), while IP-10 lower than 4271 pg/mL predicted a more favorable disease evolution (71% sensitivity, 78% specificity) (Figures 1(a) and 1(b)).

With regard to CRP, ROC curve analysis highlighted that after 7 days of hospitalization, the cut-off allowing the most accurate patients' stratification (0.79 < AUC < 0.82) was 2.3 mg/mL: CRP higher than 2.3 mg/mL predicted a worsening in clinical conditions (83% sensitivity, 73% specificity), while CRP lower than 2.3 mg/mL predicted a shorter hospitalization (81% sensitivity, 69% specificity) (Figures 2(a) and 2(b)).

ROC curves were built also for baseline values of RDW-CV, platelet count, glomerular filtration rate, and neutrophil to lymphocyte ratio (Figure 3). RDW-CV values higher than 14.3% (AUC = 0.63, 29.3% sensitivity, and 89.4% specificity) (Figure 3(a)) and platelet count lower than 192 × 10^3^/*μ*L (AUC = 0.70, 69.1% sensitivity, and 66.9% specificity) (Figure 3(b)) predicted a more severe outcome, while glomerular filtration rate higher than 75 mL/min (AUC = 0.7, 77.4% sensitivity, and 54.8% specificity) (Figure 3(c)) and neutrophil to lymphocyte ratio lower than 11.78 × 10^3^ (AUC = 0.60, 81.8% sensitivity, and 38.4% specificity) (Figure 3(d)) predicted a more favorable disease evolution.

## 4. Discussion

COVID-19 severe manifestations affect 10-20% of SARS-CoV-2-positive patients, due to a severe pneumonia depending on host's aberrant immune response [13, 15, 23]. To date, it is known that severe COVID-19 features are hypercytokinemia, hyperferritinemia, hemodynamic instability, and multiorgan failure [2, 4, 12, 13, 15, 23, 24]. Moreover, several specific abnormalities of metabolic parameters and extracellular vesicles have been observed correlated with disease refractoriness or evolution [25–28]. However, specific disease-related or severity-related accurate predictors are still lacking.

Therefore, in this observational prospective cohort, we screened a wide range of cytokines, chemokines, and routine laboratory markers to identify the best biomarkers predicting disease evolution and prognosis in hospitalized patients.

Since selected patients were hospitalized due to a moderate or severe respiratory failure, all were treated with a standardized protocol including steroids as dexamethasone or methylprednisolone and LMWH, aimed to manage the hyperinflammatory state and prothrombotic and hypercoagulable state observed in these patients [29].

By comparing the cytokine signature at hospital admission of the most severe patients to that of those experiencing milder forms of the disease, it appears that many bioactive molecules are involved (IL-4, IL-6, IL-8, IL-10, IP-10, MCP-1, MIP-1*α*, TNF-*α*, G-CSF, and IFN-*γ*); however, after multivariate statistical analysis, the only chemokine showing a clear predictive value at hospital admission is IP-10, showing both a positive association with greater disease severity and adverse prognosis, and an inverse association with faster recovery [30, 31]. Additionally, among laboratory biomarkers tested at 7 days, only CRP was predictive either of a worse or a good prognosis. IP-10 and CRP were the only laboratory parameters that retained a prognostic relevance even after correction for demographics (age and gender) and variables linked to disease severity (PiO_2_/FiO_2_ and NEWS2 score either at baseline or at 7 days) [14, 32–35].

IP-10 is an IFN-*γ* induced protein released by a large number of cells, many of which involved in the immune response, such as T cells, neutrophils, monocytes, and endothelial cells [36, 37]. IP-10 is a proinflammatory mediator involved in leukocyte homing to inflamed tissues and in the perpetuation of the inflammatory response, thus playing a pivotal role in inflammatory tissue damage [36–38]. Due to its known chemotactic action toward T cells, NK cells, monocyte/macrophages, and dendritic cells, this chemokine has been investigated as a potential biomarker for many pathological conditions (i.e., autoimmune diseases and viral infections) [36, 37]. During the 2002 SARS outbreak, many studies highlighted that higher IP-10 blood levels predicted an adverse outcome, an immunological signature also found in SARS-CoV-2 patients [31, 38–42]. In the present study, IP-10 showed a strong predictive power, with a ROC curve-based cut-off of 4271 pg/mL that, if confirmed in other studies, could be used as biomarker to identify patients needing strict clinical and therapeutic monitoring or even to drive the decision to start anti cytokine treatment.

Among the routine laboratory parameters, CRP, an acute phase protein, is known to be a strong indicator of COVID-19 severity [14, 32, 35]. The majority of the available studies confirms CRP as prognostic marker in the early stages of COVID-19 infection [43–46], even before interstitial pneumonia signs become evident at computerized tomography [47, 48]. In this study, CRP values after 7 days of hospitalization showed to have prognostic potential, with CRP higher than 2.3 mg/dL identifying patients undergoing clinical conditions worsening. Therefore, CRP value at 7 days seems to be the most reliable marker of treatment response in our population: we may speculate that the persistence of elevated CRP value at 7 days may be related to several critical conditions like the lack of corticosteroid response or eventually the development of secondary infections [44, 46, 47, 49]. Since biomarkers of treatment response in COVID-19 are lacking, to our knowledge, this result is novel, supporting the use of CRP measurement during the disease course to guide patient management.

As observed in many other studies, also for our study population, other baseline laboratory findings routinely assessed exist that contribute to a more accurate prognosis evaluation, such as RDW-CV, D-dimer levels, platelet count, and neutrophil to lymphocyte ratio at the time of hospital admission. RDW-CV is known to be a prognostic biomarker in several diseases [50–52] and also in COVID-19 patients [19, 53–55]; however, at ROC analysis, we obtained a very low sensitivity (RDW-CV values higher than 14.3% displayed 29.3% sensitivity and 89.4% specificity), therefore limiting its usefulness in clinical practice. Increased D-dimer levels and decreased platelet count were initially linked to the onset of disseminated intravascular coagulation (DIC), but today, thanks to the endlessly increase in scientific knowledge about COVID-19 disease, it is gaining attention the hypothesis of a coagulopathy with specific features different from the classical sepsis-related diffuse intravascular coagulopathy [56–58]. In this study, we observed that a baseline platelet count lower than 192 × 10^3/^*μ*L predicted a worsening in clinical conditions, while D-dimer ROC curve analysis did not allow the identification of a sufficiently accurate cut-off.

Both neutrophilia and lymphopenia are associated to COVID-19 evolution [5, 14, 21, 59, 60]. Higher neutrophil count is generally a nonspecific marker of severity, as it is related to both thromboembolic complications and systemic inflammatory responses [21], while lymphopenia is associated with a dysregulation of immune response [14]. The neutrophil to lymphocyte ratio thus magnifies the prognostic role of both events, with the ability to predict disease severity [19, 61, 62]. We confirmed a role for this biomarker since a low neutrophil to lymphocyte ratio (ROC curve-based cut − off = 11.78 × 10^3^) was associated with a shorter hospital stay.

Our study has several limitations: first, it was based on a single-center enrollment, so that a multicenter validation of our results is needed to make clinical practice recommendations. Moreover, the study was conducted in clinical practice so that slight differences in patients' treatment may have occurred; however, all patients were followed and treated according to a standardized treatment protocol issued at our center, guiding, in particular, but not limited to, steroids and heparin duration and dose, limiting the bias of different clinical approaches.

## 5. Conclusions

The increasing number of COVID-19 patients requiring hospitalization stressed the national health systems all over the world, thus highlighting the need of early predictors of disease evolution, to assist the medical staff in the patient management as well as in monitoring patients' conditions during hospitalization. In this prospective observational cohort study, we showed that, after a wide screening of different biomarkers and correction for demographic and disease severity variables, baseline IP-10 values and CRP values after 7 days hospitalization are independent predictors of patients' prognosis and in-hospital disease course and may help the physicians to stratify patients treatments.

## Figures and Tables

**Table 1:** Demographic and baseline characteristics of the studied population. ^§^ refers to data obtained with oxygen supplementation.

Demographics, parameters, and clinical scores	Median [IQR]
Age (years)	63.8 [56.2-71.9]
Heart rate (beats/min)	85 [75-95]
Respiratory rate (breaths/min)^§^	21 [18-26]
SpO_2_ (%)^§^	96 [94-98]
Temperature (°C)	36.5 [36.1-36.8]
Systolic pressure (mmHg)	125 [115-140]
Diastolic pressure (mmHg)	75 [70-85]
NEWS2	5 [4-6]
Days from illness onset to hospital admission	6 [5-8]
Laboratory findings	
Hemoglobin (g/dL)	14.2 [12.6-15.0]
RDW-CV (%)	13.3 [12.8-14.0]
White blood cells (cell count × 10^3^/*μ*L)	7.03 [5.05-9.52]
Neutrophils (cell count × 10^3^/*μ*L)	5.67 [4.20-8.56]
Eosinophils (cell count × 10^3^/*μ*L)	0.00 [0.00-0.00]
Lymphocytes (cell count × 10^3^/*μ*l)	0.71 [0.54-0.95]
Platelets (cell count × 10^3^/*μ*L)	205 [161-263]
ALT (U/L)	37 [28-55]
AST (U/L)	41 [32-57]
Bilirubin (mg/dL)	0.6 [0.5-0.8]
Creatinine (mg/dL)	0.8 [0.64-0.96]
Glomerular filtration rate (mL/min)	90 [71-103]
CRP (mg/dL)	8.2 [4.4-13.0]
LDH (U/L)	718 [554-871]
Erythrocyte sedimentation rate (mm/h)	40 [27-52]
Troponin I (ng/mL)	7 [3-15]
Ferritin (ng/mL)	826 [401-1348]
D-dimer (*μ*g/L)	697 [517-1275]
Albumin (g/dL)	4.0 [3.7-4.2]
IL-6 (pg/mL)	11.6 [5.00-31.30]
Arterial blood gas test^§^	
pO_2_ (mmHg)	70.0 [59.6-79.6]
pH	7.46 [7.44-7.49]
pCO_2_ (mmHg)	36 [33-39]
PiO_2_/FiO_2_	146 [120-180]

**Table 2:** Baseline routine laboratory findings and multiplex quantifications in patients with an adverse disease evolution (death/ICU admitted) vs. all the other patients. Data are expressed as medians (IQR). Bold text highlights the statistically significant results.

Laboratory findings at baseline (*t*0)	Adverse disease evolution (*n* = 29)	All other patients (*n* = 110)	*Z*	*p* value
Hemoglobin (g/dL)	14.3 [11.9-15.1]	14.2 [12.9-15.0]	0.3708	0.7108
RDW-CV (%)	**13.6 [13.0-14.4]**	**13.3 [12.8-13.8]**	**2.0285**	**0.0425**
White blood cells (cell count × 10^3^/*μ*L)	8.12 [5.12-9.87]	6.99 [5.05-9.21]	0.2385	0.8115
Neutrophils (cell count × 10^3^/*μ*L)	5.70 [4.20-9.15]	5.67 [4.20-7.93]	0.3940	0.6936
Eosinophils (cell count × 10^3^/*μ*L)	0.00 [0.00-0.00]	0.00 [0.00-0.00]	0.2413	0.8093
Lymphocytes (cell count × 10^3^/*μ*L)	**0.62 [0.39-0.77]**	**0.74 [5.70-0.99]**	**-2.2837**	**0.0224**
Neutrophil/lymphocyte ratio	11.90 [4.97-17.60]	7.88 [4.70-10.55]	1.7909	0.0733
Platelets (cell count × 10^3^/*μ*L)	**162 [128-200]**	**219 [176-273]**	**-4.1859**	**0.0001**
ALT (U/L)	36 [27-57]	37 [28-55]	-0.1949	0.8455
AST (U/L)	43 [36-70]	41 [31-56]	1.2971	0.1946
Bilirubin (mg/dL)	0.7 [0.5-0.8]	0.6 [0.5-0.8]	1.1019	0.2705
Creatinine (mg/dL)	**0.91 [0.77-1.24]**	**0.78 [0.64-0.90]**	**2.6236**	**0.0087**
Glomerular filtration rate (mL/min)	**75 [61-90]**	**93 [74-103]**	**-2.8489**	**0.0044**
CRP (mg/dL)	10.2 [5.6-15.5]	7.7 [4.1-12.0]	1.8531	0.0639
LDH (U/L)	**784 [694-1010]**	**690 [535-864]**	**2.1414**	**0.0322**
Erythrocyte sedimentation rate (mm/h)	44 [25-56]	40 [31-50]	0.6988	0.4847
Troponin I (ng/mL)	13 [4-17]	7 [2-14]	1.7752	0.0759
Ferritin (ng/mL)	**1155 [608-1921]**	**793 [371-1245]**	**2.2487**	**0.0245**
D-dimer (*μ*g/L)	**1175 [593-1773]**	**667 [481-1056]**	**2.2821**	**0.0225**
Albumin (g/dL)	4.0 [3.7-4.3]	4.0 [3.7-4.2]	0.6087	0.5427
Eotaxin (pg/mL)	3.45 [2.97-4.13]	3.68 [2.62-4.78]	-0.0467	0.9628
FGF (pg/mL)	12.68 [0.00-17.49]	15.72 [5.03-22.55]	-1.6272	0.1037
G-CSF (pg/mL)	61.47 [43.47-111.57]	64.27 [43.47-91.38]	-0.6610	0.5086
GM-CSF (pg/mL)	0.00 [0.00-0.00]	0.00 [0.00-0.00]	1.8897	0.0588
IFN-*γ* (pg/mL)	7.64 [5.56-9.70]	7.37 [5.20-9.98]	0.1919	0.8478
IL-1*β* (pg/mL)	0.16 [0.00-1.03]	0.53 [0.00-1.43]	-0.5697	0.5689
IL1-RA (pg/mL)	0.00 [0.00-168.65]	0.00 [0.00-168.65]	-0.2656	0.7906
IL-2 (pg/mL)	0.07 [0.00-1.79]	1.50 [0.00-4.34]	-1.8431	0.0653
IL-4 (pg/mL)	0.58 [0.00-0.90]	0.32 [0.00-0.73]	1.1959	0.2317
IL-5 (pg/mL)	0.00 [0.00-48.48]	0.00 [0.00-107.56]	-1.4608	0.1441
IL-6 (pg/mL)	**20.12 [11.51-42.41]**	**8.17 [3.8-22.45]**	**2.7546**	**0.0059**
IL-7 (pg/mL)	7.44 [1.93-19.94]	8.57 [1.93-22.56]	-0.7296	0.4656
IL-8 (pg/mL)	**22.60 [16.77-44.72]**	**15.95 [11.18-25.20]**	**2.7034**	**0.0069**
IL-9 (pg/mL)	456.18 [401.78-493.75]	456.86 [403.68-553.68]	-1.0004	0.3171
IL-10 (pg/mL)	2.05 [0.13-3.11]	1.27 [0.00-4.42]	0.4075	0.6837
IL-12 (pg/mL)	0.95 [0.00-3.23]	2.21 [0.00-4.68]	-1.8208	0.0686
IL-13 (pg/mL)	0.00 [0.00-0.49]	0.00 [0.00-0.79]	-0.1276	0.8985
IL-15 (pg/mL)	0.00 [0.00-84.08]	0.00 [0.00-273.20]	-1.0831	0.2788
IL-17 (pg/mL)	4.37 [3.65-5.95]	4.37 [2.91-6.33]	0.5474	0.5841
IP-10 (pg/mL)	**7520.77 [4953.57-12322.33]**	**3451.44 [2102.05-5850.57]**	**4.8026**	**0.0001**
MCP-1 (pg/mL)	**121.31 [67.56-203.32]**	**75.11 [40.74-103.27]**	**2.6877**	**0.0072**
MIP-1*α* (pg/mL)	1.89 [1.29-2.95]	1.69 [1.04-2.66]	0.6768	0.4985
MIP-1*β* (pg/mL)	222.66 [198.75-241.60]	228.00 [194.67-257.58]	-0.8683	0.3853
PDGF (pg/mL)	1040.46 [614.45-1910.19]	1370.46 [723.44-2622.26]	-1.4074	0.1593
RANTES (pg/mL)	5525.30 [2952.02-8016.74]	5300.72 [2982.24-10540.92]	-0.9823	0.3260
TNF-*α* (pg/mL)	19.12 [15.42-20.63]	21.91 [16.17-27.47]	-1.9106	0.0561
VEGF (pg/mL)	178.91 [19.79-230.35]	198.68 [48.82-291.68]	-1.1787	0.2385

**Table 3:** Routine laboratory findings and multiplex quantifications after 7 days hospitalization in patients with an adverse disease evolution (death/ICU admitted) vs. all the other patients. Data are expressed as medians (IQR). Bold text highlights the statistically significant results.

Laboratory findings after 7 days (*t*7)	Adverse disease evolution (*n* = 18)	All other patients (*n* = 95)	*Z*	*p* value
Hemoglobin (g/dL)	13.5 [11.8-13.9]	13.5 [12.6-14.8]	-1.3108	0.1899
RDW-CV (%)	13.4 [12.9-14.0]	13.0 [12.6-13.8]	1.3590	0.1742
White blood cells (cell count × 10^3^/*μ*L)	10.42 [6.96-13.04]	9.49 [7.52-11.02]	0.6708	0.5023
Neutrophils (cell count × 10^3^/*μ*L)	9.21 [6.18-11.81]	7.24 [5.70-8.99]	1.9144	0.0556
Eosinophils (cell count × 10^3^/*μ*L)	0.01 [0.01-0.02]	0.02 [0.01-0.08]	-1.2358	0.2165
Lymphocytes (cell count × 10^3^/*μ*L)	**0.46 [0.28-0.64]**	**1.38 [0.76-1.94]**	**-4.2958**	**0.0001**
Neutrophil/lymphocyte ratio	**19.64 [12.98-33.23]**	**6.23 [3.19-10.63]**	**4.8488**	**0.0001**
Platelets (cell count × 10^3^/*μ*L)	**295 [214-323]**	**347 [289-415]**	**-3.1070**	**0.0019**
ALT (U/L)	48 [31-130]	58 [37-98]	-0.3288	0.7423
AST (U/L)	33 [25-60]	29 [23-44]	0.9947	0.3199
Bilirubin (mg/dL)	0.8 [0.6-0.9]	0.6 [0.5-0.9]	1.0531	0.2923
Creatinine (mg/dL)	0.78 [0.60-1.09]	0.71 [0.61-0.84]	0.8046	0.4210
Glomerular filtration rate (mL/min)	93 [68-101]	97 [88-105]	-1.4266	0.1537
CRP (mg/dL)	**3.7 [2.7-9.8]**	**1.0 [0.3-2.3]**	**4.7086**	**0.0001**
LDH (U/L)	**687 [540-892]**	**575 [469-666]**	**2.7176**	**0.0066**
Erythrocyte sedimentation rate (mm/h)	**58 [41-66]**	**29 [16-46]**	**2.8219**	**0.0048**
Troponin I (ng/mL)	**6 [3-34]**	**4 [2-8]**	**2.1054**	**0.0353**
Ferritin (ng/mL)	**933 [734-1912]**	**719 [379-1017]**	**2.2958**	**0.0217**
D-dimer (*μ*g/L)	**1718 [1117-6381]**	**1001 [594-1811]**	**2.5272**	**0.0115**
Albumin (g/dL)	3.4 [3.2-3.5]	3.6 [3.4-3.8]	-1.4590	0.1446
Eotaxin (pg/mL)	4.15 [3.04-5.79]	5.07 [3.62-7.40]	-1.1539	0.2485
FGF (pg/mL)	18.09 [12.68-25.30]	21.36 [10.82-28.33]	-0.2850	0.7756
G-CSF (pg/mL)	**121.15 [89.00-192.46]**	**79.31 [61.47-103.07]**	**3.1603**	**0.0016**
GM-CSF (pg/mL)	0.00 [0.00-0.00]	0.00 [0.00-0.00]	-0.6118	0.5407
IFN-*γ* (pg/mL)	**9.70 [6.61-38.83]**	**6.83 [4.27-10.50]**	**2.2561**	**0.0241**
IL-1*β* (pg/mL)	0.16 [0.00-1.80]	0.38 [0.00-1.43]	-0.7482	0.4544
IL1-RA (pg/mL)	0.00 [0.00-135.38]	0.00 [0.00-135.38]	0.3655	0.7147
IL-2 (pg/mL)	2.01 [0.00-3.74]	0.79 [0.00-3.42]	-0.1423	0.8869
IL-4 (pg/mL)	0.32 [0.00-0.84]	0.68 [0.14-1.10]	-0.6687	0.5037
IL-5 (pg/mL)	0.00 [0.00-72.05]	0.00 [0.00-72.05]	-0.1147	0.9087
IL-6 (pg/mL)	**11.51 [9.12-28.52]**	**3.34 [0.00-8.50]**	**3.8190**	**0.0001**
IL-7 (pg/mL)	5.55 [0.00-9.00]	8.57 [0.00-22.26]	-0.7029	0.4821
IL-8 (pg/mL)	**28.59 [15.89-54.90]**	**11.02 [6.49-19.72]**	**3.6798**	**0.0002**
IL-9 (pg/mL)	460.16 [316.26-488.06]	460.82 [404.15-524.46]	-0.8905	0.3732
IL-10 (pg/mL)	1.25 [0.00-3.70]	1.25 [0.00-4.42]	-0.0189	0.9849
IL-12 (pg/mL)	0.95 [0.00-1.73]	1.58 [0.00-4.68]	-1.1909	0.2337
IL-13 (pg/mL)	0.00 [0.00-0.49]	0.00 [0.00-0.78]	-0.1755	0.8607
IL-15 (pg/mL)	0.00 [0.00-0.00]	0.00 [0.00-203.24]	-1.2231	0.2213
IL-17 (pg/mL)	3.93 [3.42-5.43]	4.87 [3.65-6.49]	-0.6190	0.5360
IP-10 (pg/mL)	**2893.33 [2672.14-6710.26]**	**737.64 [396.95-1440.44]**	**4.6502**	**0.0001**
MCP-1 (pg/mL)	**335.38 [106.19-712.15]**	**60.87 [42.11-103.27]**	**3.8958**	**0.0001**
MIP-1*α* (pg/mL)	**3.55 [2.56-5.64]**	**2.26 [1.51-3.02]**	**3.0059**	**0.0027**
MIP-1*β* (pg/mL)	230.67 [180.96-234.94]	231.62 [202.97-256.98]	-1.1378	0.2552
PDGF (pg/mL)	1622.08 [622.51-2464.00]	2270.91 [1399.66-3625.82]	-1.6820	0.0926
RANTES (pg/mL)	5444.86 [2013.72-10073.76]	7544.65 [4221.08-12495.32]	-1.2120	0.2256
TNF-*α* (pg/mL)	**18.41 [13.91-19.89]**	**24.04 [17.03-28.63]**	**-2.4685**	**0.0136**
VEGF (pg/mL)	75.81 [0.00-264.14]	119.46 [0.00-210.59]	0.0444	0.9646

**Table 4:** Multivariate analysis of baseline statistically significant routine laboratory findings and multiplex quantifications predicting an adverse disease evolution (death/ICU admission). Bold text highlights the statistically significant results.

Prognostic biomarkers (*t*0)	*β* ^∗^	*p* value
IP-10	**0.2614**	**0.0059**
Platelets	**-0.1983**	**0.0245**
D-dimer	**0.1744**	**0.0353**
RDW-CV	**0.1710**	**0.0477**
Ferritin	0.1152	0.2143
IL-6	0.1165	0.2213
MCP-1	0.1240	0.2223
IL-8	-0.0921	0.3135
Glomerular filtration rate	-0.0910	0.4844
Lymphocytes	-0.0379	0.6523
Creatinine	-0.0494	0.6850
LDH	-0.0158	0.8647

**Table 5:** Multivariate analysis of baseline statistically significant routine laboratory findings and multiplex quantifications predicting an adverse disease evolution (death/ICU admission) which were significant at multivariate analysis including demographic and COVID-19 severity-related variables. Bold text highlights the statistically significant results.

Predictors (*t*0)	*β* ^∗^	*p* value
IP-10	**0.2930**	**0.0002**
D-dimer	**0.2331**	**0.0017**
Platelets	**-0.1799**	**0.0212**
RDW-CV	**0.1508**	**0.0462**
PiO_2_/FiO_2_	-0.1269	0.0888
Age	0.1293	0.0989
Sex	-0.1031	0.1870
NEWS2	0.0795	0.2729

**Table 6:** Multivariate analysis of statistically significant routine laboratory findings and multiplex quantifications after 7 days of hospitalization. Bold text highlights the statistically significant results.

Prognostic biomarkers (*t*7)	*β* ^∗^	*p* value
CRP	**0.3772**	**0.0049**
IP-10	0.3356	0.1261
IL-6	0.4479	0.2018
LDH	-0.2065	0.2128
MCP-1	-0.5202	0.2777
IL-8	1.8435	0.2950
TNF-*α*	-1.4239	0.3563
Platelets	-0.1067	0.3914
D-dimer	-0.2360	0.4291
INF-*γ*	0.7433	0.4583
Ferritin	0.1041	0.4744
Erythrocyte sedimentation rate	0.0657	0.5932
Neutrophil/lymphocyte ratio	0.0590	0.7066
G-CSF	-0.6803	0.7070
MIP-1*α*	-0.2429	0.8606
Troponin I	-0.0182	0.8626
Lymphocytes	-0.0159	0.9039

**Table 7:** Baseline routine laboratory findings and multiplex quantifications in patients who had a faster clinical recovery (discharged or reaching NEWS2 ≤ 2 for at least 24 hours within 14 days) vs. all the other patients. Data are expressed as medians (IQR). Bold text highlights the statistically significant results.

Laboratory findings at baseline (*t*0)	Faster clinical recovery (*n* = 91)	All other patients (*n* = 48)	*Z*	*p* value
Hemoglobin (g/dL)	14.3 [12.9-15.1]	13.9 [12.4-15.0]	1.0547	0.2916
RDW-CV (%)	**13.2 [12.7-13.8]**	**13.7 [13.0-14.4]**	**-2.9173**	**0.0035**
White blood cells (cell count × 10^3^/*μ*L)	6.80 [4.91-9.09]	7.80 [5.24-10.06]	-1.2448	0.2132
Neutrophils (cell count × 10^3^/*μ*L)	5.39 [4.09-7.73]	6.50 [4.25-9.59]	-1.3910	0.1642
Eosinophils (cell count × 10^3^/*μ*L)	0.00 [0.00-0.00]	0.00 [0.00-0.00]	-0.1312	0.8956
Lymphocytes (cell count × 10^3^/*μ*L)	**0.74 [0.57-0.99]**	**0.65 [0.44-0.89]**	**1.9738**	**0.0484**
Neutrophil/lymphocyte ratio	**7.62 [4.62-10.26]**	**10.16 [5.63-16.94]**	**-2.4407**	**0.0147**
Platelets (cell count × 10^3^/*μ*L)	**219 [175-284]**	**186 [155-223]**	**2.7953**	**0.0052**
ALT (U/L)	41 [29-56]	33 [23-52]	1.7894	0.0735
AST (U/L)	42 [31-54]	41 [33-63]	-0.6809	0.4959
Bilirubin (mg/dL)	0.6 [0.5-0.8]	0.6 [0.5-0.8]	0.2432	0.8078
Creatinine (mg/dL)	**0.78 [0.62-0.88]**	**0.86 [0.67-1.13]**	**-2.5478**	**0.0108**
Glomerular filtration rate (mL/min)	**95 [78-105]**	**73 [62-91]**	**4.1183**	**<0.0001**
CRP (mg/dL)	**7.3 [4.1-11.9]**	**9.7 [5.3-15.4]**	**-2.1905**	**0.0285**
LDH (U/L)	688 [533-870]	780 [629-904]	-1.4145	0.1572
Erythrocyte sedimentation rate (mm/h)	40 [29-50]	42 [26-56]	-0.8566	0.3916
Troponin I (ng/mL)	**7 [2-13]**	**12 [4-30]**	**-2.3845**	**0.0171**
Ferritin (ng/mL)	826 [371-1278]	1032 [465-1650]	-1.1114	0.2664
D-dimer (*μ*g/L)	**660 [449-1088]**	**873 [577-1377]**	**-2.0546**	**0.0399**
Albumin (g/dL)	4.0 [3.6-4.2]	4.0 [3.8-4.2]	-0.2944	0.7685
Eotaxin (pg/mL)	3.59 [2.48-4.62]	3.57 [2.99-5.71]	-1.1962	0.2316
FGF (pg/mL)	12.68 [4.55-21.63]	16.61 [4.55-23.26]	-0.6986	0.4848
G-CSF (pg/mL)	59.83 [40.36-86.73]	71.35 [52.62-109.62]	-1.8984	0.0577
GM-CSF (pg/mL)	0.00 [0.00-0.00]	0.00 [0.00-0.00]	1.4044	0.1602
IFN-*γ* (pg/mL)	6.61 [4.73-9.59]	7.69 [5.61-11.05]	-1.6863	0.0917
IL-1*β* (pg/mL)	0.35 [0.00-1.43]	0.53 [0.14-1.84]	-0.8948	0.3709
IL1-RA (pg/mL)	0.00 [0.00-135.38]	0.00 [0.00-226.94]	-1.2088	0.2267
IL-2 (pg/mL)	1.06 [0.00-3.74]	1.06 [0.00-4.36]	-0.5243	0.6001
IL-4 (pg/mL)	**0.32 [0.00-0.68]**	**0.63 [0.00-0.99]**	**-2.1481**	**0.0317**
IL-5 (pg/mL)	0.00 [0.00-102.42]	0.00 [0.00-114.12]	-0.1084	0.9136
IL-6 (pg/mL)	**7.66 [3.13-19.51]**	**17.87 [8.17-39.87]**	**-3.3250**	**0.0009**
IL-7 (pg/mL)	7.44 [1.93-18.09]	8.57 [1.93-25.07]	-1.1357	0.2561
IL-8 (pg/mL)	**15.26 [10.84-25.05]**	**22.04 [16.50-35.79]**	**-3.6703**	**0.0002**
IL-9 (pg/mL)	446.49 [391.10-529.21]	469.28 [405.64-539.06]	-0.3455	0.7297
IL-10 (pg/mL)	**1.25 [0.00-3.91]**	**2.28 [0.52-5.67]**	**-2.1681**	**0.0302**
IL-12 (pg/mL)	2.21 [0.00-4.68]	1.65 [0.00-3.96]	0.7296	0.4657
IL-13 (pg/mL)	0.00 [0.00-0.49]	0.24 [0.00-0.68]	-1.4539	0.1460
IL-15 (pg/mL)	0.00 [0.00-229.24]	0.00 [0.00-271.68]	-0.6564	0.5116
IL-17 (pg/mL)	4.37 [2.91-5.95]	4.41 [3.33-6.20]	-0.6518	0.5145
IP-10 (pg/mL)	**3117.30 [1993.37-5384.47]**	**6964.02 [4616.95-10457.34]**	**-5.2205**	**<0.0001**
MCP-1 (pg/mL)	**73.46 [40.74-99.83]**	**110.84 [52.14-203.81]**	**-2.8661**	**0.0042**
MIP-1*α* (pg/mL)	**1.59 [0.95-2.40]**	**1.95 [1.26-3.25]**	**-2.0564**	**0.0397**
MIP-1*β* (pg/mL)	226.33 [194.67-253.07]	224.25 [193.76-263.00]	-0.2658	0.7904
PDGF (pg/mL)	1294.37 [719.67-2587.88]	1399.54 [653.99-2487.55]	0.0775	0.9382
RANTES (pg/mL)	5290.36 [3133.84-10501.29]	5729.65 [2483.56-9480.44]	0.1617	0.8716
TNF-*α* (pg/mL)	21.28 [15.42-25.97]	20.10 [15.42-25.97]	0.0709	0.9435
VEGF (pg/mL)	168.66 [0.00-273.00]	211.99 [58.45-287.78]	-0.8201	0.4121

**Table 8:** Routine laboratory findings and multiplex quantifications after 7 days of hospitalization in patients who had a faster clinical recovery (discharged or reaching NEWS2 ≤ 2 for at least 24 hours within 14 days) vs. all the other patients. Data are expressed as median (IQR). Bold text highlights the statistically significant results.

Laboratory findings after 7 days (*t*7)	Faster clinical recovery (*n* = 76)	All other patients (*n* = 37)	*Z*	*p* value
Hemoglobin (g/dL)	13.5 [12.5-15.0]	13.6 [12.3-14.2]	0.9946	0.3199
RDW-CV (%)	**13.0 [12.5-13.6]**	**13.4 [12.9-13.9]**	**-2.1164**	**0.0343**
White blood cells (cell count × 10^3^/*μ*L)	9.40 [7.22-10.91]	10.36 [8.32-12.13]	-1.7866	0.0740
Neutrophils (cell count × 10^3^/*μ*L)	**6.79 [5.59-8.37]**	**8.99 [7.22-10.55]**	**-3.3804**	**0.0007**
Eosinophils (cell count × 10^3^/*μ*L)	0.02 [0.01-0.08]	0.02 [0.00-0.04]	1.3076	0.1910
Lymphocytes (cell count × 10^3^/*μ*L)	**1.50 [0.85-1.95]**	**0.62 [0.39-1.03]**	**4.4788**	**0.0001**
Neutrophil/lymphocyte ratio	**4.82 [3.16-8.98]**	**13.19 [7.74-32.29]**	**-4.8490**	**0.0001**
Platelets (cell count × 10^3^/*μ*L)	**348 [303-417]**	**302 [260-371]**	**2.2241**	**0.0261**
ALT (U/L)	**63 [38-112]**	**41 [27-69]**	**2.4130**	**0.0158**
AST (U/L)	30 [24-45]	30 [23-40]	0.5323	0.5945
Bilirubin (mg/dL)	0.6 [0.5-0.9]	0.7 [0.6-0.9]	-0.3411	0.7330
Creatinine (mg/dL)	0.72 [0.62-0.85]	0.73 [0.55-0.83]	0.5298	0.5963
Glomerular filtration rate (mL/min)	**98 [88-107]**	**91 [69-102]**	**1.9951**	**0.0460**
CRP (mg/dL)	**0.8 [0.3-1.9]**	**3.7 [2.3-7.8]**	**-6.1875**	**<0.0001**
LDH (U/L)	**551 [449-650]**	**645 [561-819]**	**-3.3632**	**0.0008**
Erythrocyte sedimentation rate (mm/h)	**25 [16-43]**	**50 [32-63]**	**-3.1074**	**0.0019**
Troponin I (ng/mL)	**4 [2-7]**	**6 [3-22]**	**-2.2200**	**0.0264**
Ferritin (ng/mL)	751 [452-998]	793 [520-1346]	-1.2909	0.1968
D-dimer (*μ*g/L)	**903 [567-1438]**	**1755 [1132-2639]**	**-3.5792**	**0.0003**
Albumin (g/dL)	**3.6 [3.4-3.8]**	**3.4 [3.2-3.7]**	**1.9968**	**0.0458**
Eotaxin (pg/mL)	5.06 [3.59-7.92]	4.26 [3.68-6.09]	0.8166	0.4142
FGF (pg/mL)	19.63 [10.82-28.33]	20.40 [12.68-28.66]	-0.3791	0.7046
G-CSF (pg/mL)	**78.28 [59.83-98.47]**	**93.75 [77.86-156.42]**	**-2.5911**	**0.0096**
GM-CSF (pg/mL)	0.00 [0.00-0.00]	0.00 [0.00-0.00]	1.7318	0.0833
IFN-*γ* (pg/mL)	**6.14 [3.82-9.72]**	**9.57 [6.61-13.09]**	**-2.8483**	**0.0044**
IL-1*β* (pg/mL)	0.38 [0.00-1.09]	0.59 [0.00-1.43]	-0.3944	0.6933
IL1-RA (pg/mL)	0.00 [0.00-117.10]	0.00 [0.00-168.65]	-1.1670	0.2432
IL-2 (pg/mL)	0.79 [0.00-2.77]	2.01 [0.00-4.22]	-0.5259	0.5989
IL-4 (pg/mL)	0.58 [0.14-0.92]	0.68 [0.00-1.10]	-0.2517	0.8013
IL-5 (pg/mL)	0.00 [0.00-72.05]	0.00 [0.00-72.05]	0.2073	0.8357
IL-6 (pg/mL)	**2.13 [0.00-6.63]**	**9.60 [5.39-17.71]**	**-4.3785**	**0.0001**
IL-7 (pg/mL)	1.93 [0.00-18.09]	9.00 [0.91-22.56]	-1.4369	0.1507
IL-8 (pg/mL)	**9.42 [6.49-14.56]**	**21.19 [11.85-28.97]**	**-4.0087**	**0.0001**
IL-9 (pg/mL)	460.38 [403.05-520.05]	460.82 [368.17-516.36]	0.1341	0.8933
IL-10 (pg/mL)	0.97 [0.00-3.63]	2.05 [0.10-4.88]	-1.3278	0.1842
IL-12 (pg/mL)	1.34 [0.00-3.96]	1.56 [0.00-3.96]	0.0000	1.0000
IL-13 (pg/mL)	0.00 [0.00-0.79]	0.00 [0.00-0.35]	0.6089	0.5426
IL-15 (pg/mL)	0.00 [0.00-172.77]	0.00 [0.00-84.08]	0.6610	0.5029
IL-17 (pg/mL)	4.73 [3.65-6.49]	4.94 [3.42-6.37]	-0.1294	0.8970
IP-10 (pg/mL)	**668.26 [377.20-1164.56]**	**1897.80 [1022.41-4465.42]**	**-4.4249**	**0.0001**
MCP-1 (pg/mL)	**59.34 [38.81-100.05]**	**106.19 [60.87-359.83]**	**-3.2373**	**0.0012**
MIP-1*α* (pg/mL)	**2.23 [1.51-2.69]**	**3.25 [2.40-4.36]**	**-3.5158**	**0.0004**
MIP-1*β* (pg/mL)	234.90 [207.93-256.98]	230.67 [181.26-246.32]	0.9721	0.3310
PDGF (pg/mL)	2270.91 [1463.58-3558.49]	1918.40 [929.93-3491.39]	0.9099	0.3629
RANTES (pg/mL)	7255.70 [4279.12-12430.58]	7635.33 [2738.21-10391.71]	0.5172	0.6050
TNF-*α* (pg/mL)	23.77 [16.92-28.63]	19.89 [15.42-25.97]	1.1116	0.2663
VEGF (pg/mL)	105.40 [0.00-188.97]	119.46 [0.00-264.76]	-1.0418	0.2975

**Table 9:** Multivariate analysis for baseline routine laboratory findings and multiplex quantifications predicting a faster clinical recovery (hospital discharge or NEWS ≤ 2 within 14 days of hospitalization). Bold text highlights the statistically significant results.

Prognostic biomarkers (*t*0)	*β* ^∗^	*p* value
Glomerular filtration rate	**0.3828**	**0.0046**
IP-10	**-0.2388**	**0.0226**
Neutrophil/lymphocyte ratio	**-0.2033**	**0.0138**
Creatinine	0.2719	0.1363
RDW-CV	-0.1229	0.1524
D-dimer	-0.1115	0.2090
IL-6	-0.1222	0.1952
MIP-1*α*	0.2804	0.1768
MCP-1	-0.0815	0.4383
Troponin I	-0.0861	0.5313
IL-8	-0.1307	0.3177
Platelets	0.0731	0.3932
IL-10	-0.1754	0.5810
CRP	-0.0061	0.9450
Lymphocytes	-0.0011	0.9882
IL-4	0.0668	0.8293

**Table 10:** Multivariate analysis for baseline routine laboratory findings and multiplex quantifications predicting a faster clinical recovery (hospital discharge or NEWS ≤ 2 within 14 days of hospitalization) including demographic and COVID-19 severity-related variables. Bold text highlights the statistically significant results.

Predictors (*t*0)	*β* ^∗^	*p* value
IP-10	**-0.2999**	**0.0004**
Age	**-0.2653**	**0.0076**
PiO_2_/FiO_2_	**0.1990**	**0.0085**
Neutrophil/lymphocyte ratio	-0.1490	0.4120
NEWS2	-0.0590	0.0602
Sex	-0.0293	0.7031
Glomerular filtration rate	-0.0168	0.8723

**Table 11:** Multivariate statistical analysis for routine laboratory findings and multiplex quantifications predicting a faster clinical recovery (hospital discharge or NEWS ≤ 2 within 14 days of hospitalization) after 7 days of hospitalization. Bold text highlights the statistically significant results.

Prognostic biomarkers (*t*7)	*β* ^∗^	*p* value
CRP	**-0.4455**	**0.0024**
Glomerular filtration rate	0.2647	0.1159
G-CSF	3.8136	0.0612
Troponin I	0.1827	0.1591
MIP-1*α*	-2.5994	0.1009
LDH	0.2445	0.1679
IL-8	-1.2701	0.2966
IP-10	-0.2288	0.2800
Neutrophils	-0.1053	0.5295
D-dimer	-0.3139	0.3026
ALT	-0.0981	0.4299
MCP-1	0.3322	0.3968
IL-6	0.2213	0.5519
Platelets	0.0784	0.5607
Lymphocytes	0.0239	0.8613
Neutrophil/lymphocyte ratio	-0.1745	0.4233
Albumin	0.0452	0.7511
Erythrocyte sedimentation rate	0.0770	0.5592
IFN-*γ*	0.0536	0.9560
RDW-CV	-0.0024	0.8355

## Data Availability

Data are available upon reasonable request to be addressed to the corresponding author.

## References

[1] Gomez-Mesa J. E., Galindo-Coral S., Montes M. C., Muñoz Martin A. J. (2021). Thrombosis and coagulopathy in COVID-19. *Current Problems in Cardiology*.

[2] Hu B., Huang S., Yin L. (2021). The cytokine storm and COVID-19. *Journal of Medical Virology*.

[3] Bellan M., Gavelli F., Hayden E. (2021). Pattern of emergency department referral during the Covid-19 outbreak in Italy. *Panminerva Medica*.

[4] Hu B., Guo H., Zhou P., Shi Z.-L. (2021). Characteristics of SARS-CoV-2 and COVID-19. *Nature Reviews Microbiology*.

[5] Harrison A. G., Lin T., Wang P. (2020). Mechanisms of SARS-CoV-2 transmission and pathogenesis. *Tends in Immunology*.

[6] Meyerowitz E. A., Richterman A., Gandhi R. T., Sax P. E. (2021). Transmission of SARS-CoV-2: a review of viral, host, and environmental factors. *Annals of Internal Medicine*.

[7] Sanyal S. (2020). How SARS-CoV-2 (COVID-19) spreads within infected hosts – what we know so far. *Emerging Topics in Life Sciences*.

[8] Rutter H., Parker S., Stahl-Timmins W. (2021). Visualising SARS-CoV-2 transmission routes and mitigations. *BMJ*.

[9] Buitrago-Garcia D., Egli-Gany D., Counotte M. J. (2020). Occurrence and transmission potential of asymptomatic and presymptomatic SARS-CoV-2 infections: a living systematic review and meta-analysis. *PLoS Medicine*.

[10] Oran D. P., Topol E. J. (2020). Prevalence of asymptomatic SARS-CoV-2 infection. A narrative review. *Annals of Internal Medicine*.

[11] Bellan M., Sainaghi P. P., Gavelli F. (2020). Lessons from the Italian COVID-19 frontline. *Minerva Medica*.

[12] Bhaskar S., Sinha A., Banach M. (2020). Cytokine storm in COVID-19 – immunopathological mechanisms, clinical considerations and therapeutic approaches: the REPROGRAM consortium position paper. *Frontiers in Immunology*.

[13] Coperchini F., Chiovato L., Croce L., Magri F., Rotondi M. (2020). The cytokine storm in COVID-19: an overview of the involvement of the chemokine/chemokine-receptor system. *Cytokine and Growth Factor Reviews*.

[14] Ding L., Zhang W., Zhang F. (2021). Prognostic role and diagnostic power of seven indicators in COVID-19 patients. *Frontiers in Medicine*.

[15] Cabaro S., D’Esposito V., Di Matola T. (2021). Cytokine signature and COVID-19 prediction models in the two waves of pandemics. *Scientific Reports*.

[16] Polverino F., Stern D. A., Ruocco G. (2020). Comorbidities, cardiovascular therapies, and COVID-19 mortality: a nationwide, Italian observational study (ItaliCO). *Frontiers in cardiovascular medicine*.

[17] Bellan M., Patti G., Hayden E. (2020). Fatality rate and predictors of mortality in an Italian cohort of hospitalized COVID-19 patients. *Scientific reports*.

[18] Pinato D. J., AJX L., Biello F. (2020). Presenting features and early mortality from SARS-CoV-2 infection in cancer patients during the initial stage of the COVID-19 pandemic in Europe. *Cancers*.

[19] Bellan M., Azzolina D., Hayden E. (2021). Simple parameters from complete blood count predict in-hospital mortality in COVID-19. *Disease Markers*.

[20] Corradini E., Ventura P., Ageno W. (2021). Clinical factors associated with death in 3044 COVID-19 patients managed in internal medicine wards in Italy: results from the SIMI-COVID-19 study of the Italian Society of Internal Medicine (SIMI). *Internal and emergency medicine*.

[21] Li J., He X., Yuan Y. (2021). Meta-analysis investigating the relationship between clinical features, outcomes, and severity of severe acute respiratory syndrome coronavirus 2 (SARS-CoV-2) pneumonia. *American Journal of Infection Control*.

[22] Gidari A., De Socio G. V., Sabbatini S., Francisci D. (2020). Predictive value of National Early Warning Score 2 (NEWS2) for intensive care unit admission in patients with SARS-CoV-2 infection. *Infectious Diseases*.

[23] Shcherbak S. G., Anisenkova A. Y., Mosenko S. V. (2021). Basic predictive risk factors for cytokine storms in COVID-19 patients. *Frontiers in Immunology*.

[24] Castelli V., Cimini A., Ferri C. (2020). Cytokine storm in COVID-19: “when you come out of the storm, you won’t be the same person who walked in”. *Frontiers in Immunology*.

[25] Barberis E., Timo S., Amede E. (2020). Large-scale plasma analysis revealed new mechanisms and molecules associated with the host response to SARS-CoV-2. *International Journal of Molecular Sciences*.

[26] Barberis E., Amede E., Tavecchia M. (2021). Understanding protection from SARS-CoV-2 using metabolomics. *Scientific Reports*.

[27] Barberis E., Vanella V. V., Falasca M. (2021). Circulating exosomes are strongly involved in SARS-CoV-2 infection. *Frontiers in Molecular Biosciences*.

[28] Cappellano G., Raineri D., Rolla R. (2021). Circulating platelet-derived extracellular vesicles are a hallmark of Sars-Cov-2 infection. *Cell*.

[29] Braz-de-Melo H. A., Faria S. S., Pasquarelli-do-Nascimento G., Santos I. O., Kobinger G. P., Magalhes K. G. (2021). The use of the anticoagulant heparin and corticosteroid dexamethasone as prominent treatments for COVID-19. *Frontiers in Medicine*.

[30] Lev S., Fottesman T., Levin G. A. (2021). Observational cohort study of IP-10’s potential as a biomarker to aid in inflammation regulation within a clinical decision support protocol for patients with severe COVID-19. *PLoS One*.

[31] Yang Y., Shen C., Li J. (2020). Plasma IP-10 and MCP-3 levels are highly associated with disease severity and predict the progression of COVID-19. *The Journal of Allergy and Clinical Immunology*.

[32] Mosquera-Sulbaran J. A., Pedreañez A., Carrero Y., Callejas D. (2021). C-reactive protein as an effector molecule in Covid-19 pathogenesis. *Reviews in Medical Virology*.

[33] Howe H. S., Ling L. M., Elangovan E. (2021). Plasma IP-10 could identify early lung disease in severe COVID-19 patients. *Annals of the Academy of Medicine, Singapore*.

[34] Ali A. M., Rostam H. M., Fatah M. H., Noori C. M., Ali K. M., Tawfeeq H. M. (2021). Serum troponin, D-dimer, and CRP level in severe coronavirus (COVID-19) patients. *Immunity, Inflammation and Disease*.

[35] Yitbarek G. Y., Ayehu G. W., Asnakew S. (2021). The role of C-reactive protein in predicting the severity of COVID-19 disease: a systematic review. *SAGE Open Medicine*.

[36] Antonelli A., Ferrari S. M., Giuggioli D., Ferrannini E., Ferri C., Fallahi P. (2014). Chemokine (C-X-C motif) ligand (CXCL)10 in autoimmune diseases. *Autoimmunity Reviews*.

[37] Lee E. Y., Lee Z. H., Song Y. W. (2013). The interaction between CXCL10 and cytokines in chronic inflammatory arthritis. *Autoimmunity Reviews*.

[38] Chen Y., Wang J., Liu C. (2020). IP-10 and MCP-1 as biomarkers associated with disease severity of COVID-19. *Molecular Medicine*.

[39] Tripathy A. S., Vishwakarma S., Trimbake D. (2021). Pro-inflammatory CXCL-10, TNF-*α*, IL-1*β*, and IL-6: biomarkers of SARS-CoV-2 infection. *Archives of Virology*.

[40] Liu Y., Zhang C., Huang F. (2020). Elevated plasma levels of selective cytokines in COVID-19 patients reflect viral load and lung injury. *National Science Review*.

[41] Velavan T. P., Meyer C. G. (2020). Mild versus severe COVID-19: laboratory markers. *International Journal of Infectious Diseases*.

[42] Chen J., Subbarao K. (2007). The immunobiology of SARS. *Annual Review of Immunology*.

[43] Stringer D., Braude P., Myint P. (2021). The role of C-reactive protein as a prognostic marker in COVID-19. *International Journal of Epidemiology*.

[44] Wang L. (2020). C-reactive protein levels in the early stage of COVID-19. *Médecine et Maladies Infectieuses*.

[45] Luo X., Zhou W., Yan X. (2020). Prognostic value of C-reactive protein in patients with coronavirus 2019. *Clinical Infectious Diseases*.

[46] Chen W., Zheng K. I., Liu Z., Yan Z., Xu C., Qiao Z. (2020). Plasma CRP level is positively associated with the severity of COVID-19. *Annals of Clinical Microbiology and Antimicrobials*.

[47] Luan Y.-Y., Yin C.-H., Yao Y. M., Yao Y.-M. (2021). Update advances on C-reactive protein in COVID-19 and other viral infections. *Frontiers in Immunology*.

[48] Tan C., Huang Y., Shi F. (2020). C-reactive protein correlates with computed tomographic findings and predicts severe COVID-19 early. *Journal of Medical Virology*.

[49] Sahu B. R., Kampa R. K., Padhi A., Panda A. K. (2020). C-reactive protein: a promising biomarker for poor prognosis in COVID-19 infection. *Clinica Chimica Acta*.

[50] Bellan M., Soddu D., Zecca E. (2020). Association between red cell distribution width and response to methotrexate in rheumatoid arthritis. *Reumatismo*.

[51] Bellan M., Giubertoni A., Piccinino C. (2019). Red cell distribution width and platelet count as biomarkers of pulmonary arterial hypertension in patients with connective tissue disorders. *Disease Markers*.

[52] Soddu D., Sola D., Bellan M. (2021). Red cell distribution width is a potential predictor of early relapse in polymyalgia rheumatica. *Reumatismo*.

[53] Soni M., Gopalakrishnan R. (2021). Significance of RDW in predicting mortality in COVID‐19—an analysis of 622 cases. *International Journal of Laboratory Hematology*.

[54] Lee J. J., Montazerin S. M., Jamil A. (2021). Association between red blood cell distribution width and mortality and severity among patients with COVID-19: a systematic review and meta-analysis. *Journal of Medical Virology*.

[55] Banon T., Wortsman J., Moshe S. B. (2021). Evaluating red blood cell distribution width from community blood tests as a predictor of hospitalization and mortality in adults with SARS-CoV-2: a cohort study. *Annals of Medicine*.

[56] Émile C. (2021). Risque thrombotique de la Covid-19. *OptionBio*.

[57] Guglielmetti G., Quaglia M., Sainaghi P. P. (2020). "War to the knife" against thromboinflammation to protect endothelial function of COVID-19 patients. *Critical Care*.

[58] Helms J., Tacquard C., Severac F. (2020). High risk of thrombosis in patients with severe SARS-CoV-2 infection: a multicenter prospective cohort study. *Intensive care medicine*.

[59] Li S., Jiang L., Li X. (2020). Clinical and pathological investigation of patients with severe COVID-19. *JCI Insight*.

[60] Liu J., Li S., Liu J. (2020). Longitudinal characteristics of lymphocyte responses and cytokine profiles in the peripheral blood of SARS-CoV-2 infected patients. *EBioMedicine*.

[61] Zheng Z.-Y., Feng S. D., Chen G.-P., Wu J.-N. (2021). Predictive value of the neutrophil to lymphocyte ratio for disease deterioration and serious adverse outcomes in patients with COVID-19: a prospective cohort study. *BMC Infectious Diseases*.

[62] Simadibrata D. M., Calvin J., Wijaya A. D., Ibrahim N. A. A. (2021). Neutrophil-to-lymphocyte ratio on admission to predict the severity and mortality of COVID-19 patients: a meta-analysis. *American Journal of Emergency Medicine*.

